# Characterization of Coffee Waste-Based Biopolymer Composite Blends for Packaging Development

**DOI:** 10.3390/foods14111991

**Published:** 2025-06-05

**Authors:** Gonzalo Hernández-López, Laura Leticia Barrera-Necha, Silvia Bautista-Baños, Mónica Hernández-López, Odilia Pérez-Camacho, José Jesús Benítez-Jiménez, José Luis Acosta-Rodríguez, Zormy Nacary Correa-Pacheco

**Affiliations:** 1 Centro de Desarrollo de Productos Bióticos, Instituto Politécnico Nacional, Carretera Yautepec-Jojutla km. 6, Calle CEPROBI No. 8. Col. San Isidro, Yautepec C.P. 62731, Morelos, Mexico; ghernandezl1700@alumno.ipn.mx (G.H.-L.); lbarrera@ipn.mx (L.L.B.-N.); sbautis@ipn.mx (S.B.-B.); 2 Centro de Investigación en Química Aplicada (CIQA), Blvd. Enrique Reyna No. 140, Col. San José de los Cerritos, Saltillo C.P. 25294, Coahuila, Mexico; odilia.perez@ciqa.edu.mx; 3 Instituto de Ciencia de Materiales de Sevilla, CSIC-Universidad de Sevilla, Avda. Américo Vespucio 49, Isla de la Cartuja, 41092 Sevilla, Spain; benitez@icmse.csic.es; 4 Centro Interdisciplinario de Investigación para el Desarrollo Integral Regional, Unidad Sinaloa, Instituto Politécnico Nacional, Boulevard Juan de Dios Bátiz 250, Guasave C.P. 81049, Sinaloa, Mexico; jlacostar@ipn.mx

**Keywords:** agricultural waste, functionalization, nanoparticles, coffee, chitosan, coating

## Abstract

In recent years, coffee waste by-products have been incorporated into polymer blends to reduce environmental pollution. In this study, coffee parchment (CP) was incorporated into biodegradable polylactic acid (PLA) and poly (butylene adipate-co-terephthalate) (PBAT) polymer blends to prepare ribbons through the extrusion process. Extracted green coffee bean oil (CO) was used as a plasticizer, and CP was used as a filler with and without functionalization. A solution of chitosan nanoparticles (ChNp) as a coating was applied to the ribbons. For the raw material, proximal analysis of the CP showed cellulose and lignin contents of 53.09 ± 3.42% and 23.60 ± 1.74%, respectively. The morphology of the blends was observed via scanning electron microscopy (SEM). Thermogravimetric analysis (TGA) showed an increase in the ribbons’ thermal stability with the functionalization. The results of differential scanning calorimetry (DSC) revealed better miscibility for the functionalized samples. The mechanical properties showed that with CP incorporation into the blends and with the ChNp coating, the Young’s modulus and the tensile strength decreased with no significant changes in the elongation at break. This work highlights the potential of reusing different by-products from the coffee industry, such as coffee oil from green beans and coffee parchment as a filler, and incorporating them into PLA PBAT biodegradable polymer blend ribbons with a nanostructured antimicrobial coating based on chitosan for future applications in food packaging.

## 1. Introduction

Coffee is the second most consumed drink around the world after water, and due to the scale of its production, coffee farming generates a high amount of waste. Coffee’s worldwide popularity has led to a harvested area of 12,205,702 ha, the production of 10,782,333.89 tons per year, and gross production of USD 15,040,064 [1]. As a result, it is necessary to generate a more sustainable process and use for these by-products [2]. Coffee cherries on a dry basis are composed of 9.9% peels and pulp, 1.7% mucilage, 4.1% silver skin and parchment, 19% grain, and 65.3% water [3]. One of the potential uses of coffee residues is the development of biodegradable polymer composites to generate new environmentally friendly alternatives [4]. Today, there are several polymers obtained from natural resources, as well as biocomposites based on coffee by-products such as bagasse and silver skin, which are lignocellulosic residues rich in functional compounds used in the elaboration of intelligent packaging [5,6]. Most of the polymers employed for packaging, such as polyethylene terephthalate, polystyrene, and polypropylene, are of synthetic origin, generating a negative impact on the environment and health. Therefore, biodegradable and sustainable polymers of natural origins are of special interest [7]. Polylactic acid (PLA) is a biodegradable polymer of high compatibility and biodegradability [8,9]. On the other hand, poly (butylene adipate-co-terephthalate) (PBAT) is a synthetic, biodegradable and compostable polymer obtained from fossil-based resources [10,11]. There are many reports on PLA and PBAT blends due to their compatibility [12]. However, to improve the physical characteristics of polymer blends, functionalization is employed. One method of functionalization is the reduction of hydroxyl groups, which consists of the sample’s acetylation, to improve compatibility between polymers [13,14,15,16]. On the other hand, plasticizers improve rheological characteristics and increase the material’s flexibility [17]. Today, plasticizers of natural origin are commonly used because they have low toxicity, they can be easily incorporated into the environment, and they are readily available. Vegetable oils such as soybean, linseed, castor, and sunflower are used as natural plasticizers [18]. There are many studies in which coffee residues have been incorporated into polymers, such as coffee grounds obtained from espresso coffee machines to produce biodegradable films based on polybutylene succinate [19] and the production of composites with the addition of coffee peel and husk particles into polyhydroxybutyric acid [20]. Moreover, it has been reported that the oil obtained from spent coffee grounds has been used as a plasticizer in PLA-based composites, improving the compatibility of the polymer blend in addition to achieving good performance for physical properties such as strength and flexibility [21]. Lule and Kim [22] prepared compounds based on PBAT and coffee husks. They modified the coffee husks to improve compatibility with the polymer, resulting in an increase in the mechanical properties. On the other hand, chitosan is a natural polymer derived from chitin, which is an abundant polysaccharide, having several properties such as non-toxicity, biocompatibility, and biodegradability [23]. Nanostructured coatings, in which nanometric scale compounds are incorporated, have been proven to lengthen the shelf-life of food, because these compounds act as antimicrobial agents in intelligent packaging [24,25]. Using nanotechnology in packaging can bring about many advantages, like improving the transport of active substances and enhancing bioavailability, physicochemical stability of the packaging composition, and antimicrobial properties [26]. Hernández-López et al. [27] found that the use of chitosan as a coating in polymer fibers enhanced the mechanical properties of the PLA and PBAT fibers by stiffening them.

Regarding the use of coffee residues in postharvest applications, Andrea et al. [28] assessed the antifungal efficacy of coffee ground extract and ChNp for controlling the fungus *Rhizopus stolonifer*. They developed, characterized, and applied two nanostructured edible coatings (ChNp and coffee ground extract encapsulated in ChNp) on Naples tomatoes, evaluating their fungicidal activity and their effect on fruit quality. A synergistic effect was observed between the ChNp and the 1% coffee ground extract, with higher inhibition of fungi mycelial growth (43%). Disease severity also decreased by 33% for the coated tomatoes. For the fruit quality variables, differences were observed in color and CO_2_ production. Divyashri et al. [29] investigated the effects of two edible coatings composed of clove oil and coffee husk pectin on extending the shelf-life of grapes during post-harvest storage. The results showed that the coated grapes maintained higher quality compared with the uncoated ones, and with a freeze-dried coating, the weight loss decreased by up to 76% under ambient conditions, showing *in vitro* inhibition against *Staphylococcus aureus*. The coated grapes showed higher retention of bioactive compounds, with total phenolic and flavonoid retention of 86.9% and 83.7%, respectively, maintaining their quality and safety during post-harvest storage. Vallejos-Jiménez et al. (2025) [30] developed a pectin-based film reinforced with bacterial cellulose obtained through the fermentation of coffee mucilage and coffee oil extract from coffee grounds, showing the enhanced properties of the reinforced films based on coffee pulp pectin compared with films based on coffee mucilage pectin (tensile strength of 2.2 MPa and water solubility of 58.5%). This effect was attributed to the morphological characteristics and the presence of pores in the polymer matrix of the coffee mucilage pectin-based films, yielding promising substitutes for single-use plastics. The objective of this work was to reuse the coffee residues generated by industry, such as CP and coffee oil extracted from green beans, and incorporate them into a biodegradable packaging. The significance lies in the revalorization of coffee residues, transforming them into a valuable resource and contributing to a circular economy. Also, it contributes to reducing the actual main pollution problem caused by synthetic packaging. The aim of this study was the development of an innovative, sustainable packaging material including biopolymers, coffee waste, and a nanostructured coating using an antimicrobial natural polymer such as chitosan for applications in postharvest horticultural product preservation.

## 2. Materials and Methods

### 2.1. Coffee Parchment Conditioning

The CP was provided by coffee producers in Coatepec, Veracruz, Mexico. The residues were cleaned and separated manually to eliminate external agents. Subsequently, they were oven-dried (Binder^®^ FD23ULE2, Tuttlingen, Germany) at 60 °C for 12 h. After drying, the sample was ground (INMIMEX M-150, Tlaxcala, Mexico). The recovered powder particles were homogenized with a #50 sieve mesh (100 µm sieve size) and then stored at room temperature for further use.

### 2.2. Coffee Parchment Characterization

#### 2.2.1. Moisture Content

The moisture content was determined using the method described by the Asociation of Analytical Communities (AOAC) [31], which consists of weighing 3 g of sample powder and placing them in a vacuum drying oven (Yamato ADP200C, Santa Clara, CA, USA) at 105 °C for 3 h. They were weighed on an analytical balance. The moisture percentage was calculated as follows:

(1) $$\%\;moisture=\frac{Iw-Fw}{Iw}\times 100,$$

Here, *Iw* is the initial sample weight, and *Fw* is the final sample weight.

#### 2.2.2. Ash Content

The ash determination was carried out with the AOAC method [31]. For this, 2 g of CP dry powder was placed in porcelain crucibles in a muffle (Thermolyne Furnace 1400, Buffalo, NY, USA) at 600 °C for 4 h to calcinate the material and obtain ash. At the end of the calcination, the crucibles were left to cool in a desiccator and weighed on an analytical balance. The percentage of ash was obtained using Equations (2)–(4):

(2) $$SW=CWS\left(g\right)-ECW(g),$$

Here, *SW* is the sample weight (g), *CWS* is the crucible weight with the sample (g), and *ECW* is the empty crucible weight, while in

(3) $$AW=CWS\left(g\right)-ECW(g),$$

*AW* is the ash weight, and

(4) $$\%\;Ash=\frac{AW}{SW}\times 100,$$

#### 2.2.3. Cellulose Content

For cellulose determination, 0.5 g of CP powder was weighed, mixed in a solution of ethanol and concentrated nitric acid (4:1 *v*/*v*), and refluxed in a cold-water bath for 1 h. The obtained residue was washed with hot distilled water for 1 h and, after that, with a saturated sodium acetate solution. At the end of the process, hot distilled water was added again. Then, the sample was filtered, the residue was oven-dried (Binder^®^ FD23ULE2, Tuttlingen, Germany) at 105 °C, and the weight was recorded. The percentage of cellulose was calculated using the following equation:

(5) $$\%\;Cellulose=\frac{DWR}{WCM}\times 100,$$

where *DWR* is the dry weight of the residue and *WCM* is the weight of the cellulosic material.

#### 2.2.4. Lignin Content

The lignin content was measured by adding 100 mg of CP and 10 mL of 72% H_2_SO_4_ to an Erlenmeyer flask. The mixture was constantly stirred for 2 h at a temperature of 50 °C. Then, 170 mL of distilled water was added to the sample, which was placed in an autoclave at 120 °C and 1.05 bar for 1 h. The material was allowed to cool and filter through a polyester membrane using hot water. Finally, the samples were dried in an oven at 60 °C for 12 h, and the weight was recorded. The percentage of lignin was calculated using Equation (6):

(6) $$\%\;Lignin=\frac{LW}{DSW}\times 100,$$

where *LW* is the lignin weight and *DSW* is the dry sample weight.

#### 2.2.5. Protein Content

Protein determination was performed using the Kjeldahl method based on the American Association of Cereal Chemists (AACC) 46-13 method [32]. The protein percentage was determined by total nitrogen quantification, which consisted of weighing 1 g of the sample and placing it in a Kjendahl tube, to which 1 g of copper sulfate, 10 g of anhydrous potassium sulfate, and 15 mL of concentrated sulfuric acid were added. The sample was placed in a digester and gradually heated to 400 °C until the tube contents turned a light blue-green color. The tube was allowed to cool to room temperature. Subsequently, 15 mL of distilled water was used for washing, and 50 mL of 40% sodium hydroxide was added. Then, 50 mL of 4% boric acid and 5 drops of Wesslow indicator were added to a flask. Distillation was carried out until obtaining a volume of 100 mL. The obtained sample was titrated with 0.1 N hydrochloric acid. The percentage of protein was calculated using Equations (7) and (8):

(7) $$\%\;Protein=\frac{mL\;spent\times N\times 14}{sample\;weight\times 1000}\times F\times 100,$$

(8) $$\%\;Available\;nitrogen=\frac{mL\;spent\times mN\times 1.4007}{sample\;weight}\times F,$$

where *F* is the protein factor (6.25), *N* is the titration normality (0.1), and *mN* is the milliequivalents of nitrogen (0.014).

#### 2.2.6. Fat Content

Soxhlet extraction was used for the fat measurement, with petroleum ether as the solvent and the plant material used on a dry basis. First, the distillation flasks, the distillation cartridges, and the powder at a constant weight were placed into the reflux system in the Soxhlet apparatus. Approximately 3.5 g of the sample was placed in each cartridge and left for 6 h in the reflux system with petroleum ether separately. Finally, the samples were removed, and the cartridges and flasks were placed in a desiccator to record the weight. All analyses were carried out in triplicate. The percentage of crude fat on a dry basis was determined using Equations (9) and (10):

(9) FW=WCF−ECW,

(10) $$\%CFdb=\frac{FW}{SW}\times 100,$$

where %*CFdb* is the crude fat percentage on dry basis, *FW* is the fat weight, and *SW* is the sample weight.

### 2.3. Green Coffee Bean Oil Extraction

To obtain the oil (CO), the green coffee beans were ground and sieved through a #60 mesh. Hexane was used as the solvent. In the Soxhlet reflux system, 50 g of the sample was placed in the cartridge and left for 6 h. Then, the extracted oil was recovered and further concentrated in a rotary evaporator (BUCHI R-300, Flawil, Switzerland) to eliminate solvent residues. Finally, the oil was packaged and stored in a refrigerator.

### 2.4. CP Functionalization

First, the CP was functionalized using the method proposed by Zhang et al. [13]. For this, 10 g of dry sample were acetylated by adding 10 mL of glacial acetic acid, followed by 30 mL of anhydrous acetic acid and finally 0.38 mL of 98% sulfuric acid diluted in 10 mL of glacial acetic acid for 90 min at 70 °C. At the end, excess acid was removed with distilled water, followed by drying in a vacuum oven (Yamato, model ADP200C, Tokyo, Japan) for 24 h.

### 2.5. Polymer Blend Preparation

The CP and the polymers were blended using an internal mixer (Brabender^®^ Instruments, Duisburg, Germany), in which CP (5, 7.5, and 10%) with and without functionalization was incorporated into a PLA PBAT 60/40 blend. The blending conditions were 60 rpm at 200 °C for 10 min with a speed rotor (CAM type) rotating at 60 rpm. The samples were dried prior to the extrusion process and pelletized using a hammer mill (Brabender^®^ Instruments, Duisburg, Germany) with a 0.5 mm sieve.

After that, a twin-screw extruder (Process 11, Thermo Scientific^TM^, Waltham, MA, USA) was used for ribbon preparation with the PLA PBAT 60/40 polymer matrix. The barrel extruder’s operating conditions were 160/180/180/180/180/170/170/150 °C, and for the die, it was 160 °C. The PLA, PBAT pellets, and CP were previously dried in a vacuum oven (Yamato, model ADP200C, Tokyo, Japan) at 60 °C for 24 h for moisture removal. The CO was incorporated through the second feed port with the aid of a peristaltic pump (MasterFlex C/L, Cole-Parmer, Vernon Hills, IL, USA) at an injection speed of 0.2 rpm. The proportion of oil that was used for each treatment was 2 g of oil for every 30 g of polymer blends. At the end of extrusion, the blend was cooled with water. For the nanostructured chitosan coating, the obtained ribbons were cooled with a solution containing chitosan and ChNp. Chitosan (América Alimentos, Zapopan, Jal., Mexico) at 1% *w*/*v* with a molecular weight of 89.3 kDa and a degree of deacetylation of 89.4% was employed to prepare the coating solution. The ratio of chitosan solution to chitosan nanoparticles was 9:1. The chitosan coating was prepared at 1% *w*/*v* by dissolving 20 g in 2000 mL of distilled water with 1% commercial vinegar under constant stirring for 24 h while adjusting the pH to 5.6 with 1N NaOH. To synthesize the ChNp, 0.05% chitosan *w*/*v* was added to 96% ethanol into a flask with the aid of a peristaltic pump (MasterFlex C/L, Thermo Fisher Scientific, Vernon Hills, IL, USA) under constant stirring. After that, the obtained solution was concentrated using a rotary evaporator (Buchi R-300, Flawil, Switzerland). Finally, the obtained concentrated nanoparticles (200 mL) were mixed with 1800 mL of 1% chitosan with the aid of a homogenizer (Virtis, Gardiner, NY, USA) at 10,000 rpm for 30 min for further use in the extrusion cooling bath. The different formulations are shown in Table 1.

### 2.6. Sample Characterization

#### 2.6.1. SEM

SEM was performed to observe the morphology of the blends and CP. For this, a cryogenic fracture was performed on the ribbons using liquid nitrogen. After that, the samples were coated in gold. The micrographs were obtained using an electron microscope (Tabletop Microscope TM-1000, Hitachi, Tokyo, Japan) with an accelerating voltage of 15 kV at different magnifications.

#### 2.6.2. FTIR

The CP, CO, and polymer blend functional groups were identified using confocal micro-Raman spectroscopy coupled with Fourier transform infrared-attenuated total reflection (FTIR-ATR). The FTIR was conducted using a FTIR equipment (Horiba Jobin Yvon, model IR2, Edison, NJ, USA), with a wavelength of 4000–400 cm^−1^, a DTGS detector performing 32 scans, and a resolution of 4 cm^−1^.

#### 2.6.3. TGA

TGA measurement for the CP, CO, and polymer blends was conducted using DSC Discovery Series equipment (TA Instruments, New Castle, DE, USA). For each test, 0.5 ± 0.1 g of the sample was placed in a platinum crucible and subjected to a temperature rate of 10 °C/min from 20 to 500 °C under an inert nitrogen atmosphere. The data obtained were analyzed using TRIOS software Version 5.1.

#### 2.6.4. DSC

Changes in the thermal transitions of the samples were obtained using a calorimeter (DSC Q20) under a nitrogen atmosphere with a heating rate of 10 °C/min from 20 °C to 170 °C and a temperature of −70 °C for the cooling stage. The sample weight was 0.5 ± 0.1 g. The results were calculated using Universal Analysis 2000 software (Version 5.1, TA Instruments, New Castle, DE, USA).

#### 2.6.5. Mechanical Properties

The tensile properties were determined using a universal testing machine (MTS Criterion, model 42, Eden Prairie, MN, USA) according to the ASTM D638-03 standard test [33]. Ten specimens were measured. Tests were carried out at room temperature (25 °C ± 1) with 10 repetitions for each treatment. A 250 N load cell was used with a speed of 1 mm/min.

### 2.7. Statistical Analysis

Statistical analysis was performed using InfoStat software version 2020 with one-way analysis of variance (ANOVA) with Duncan’s multiple range test.

## 3. Results and Discussion

### 3.1. Coffee Parchment Proximal Analysis

Table 2 shows that the CP powder had a 3.25 ± 0.17% moisture content, similar to the value reported by Chala et al. [34] for 96% dry matter (4% moisture). The ash content was found to be 0.71 ± 0.03% within the parameters of 0.5–1% reported by Esquivel and Jiménez [35]. However, a lower value was found by Wondemagegnehu et al. [36], while Mendoza Martinez et al. [37] reported an ash content in the range of 5.84–7.17%.

A cellulose content of 53.09 ± 3.42% was determined, similar to the value of 41.2% reported by Mendoza Martinez et al. [38] for the CP. For the lignin content, a value of 23.60 ± 1.74% was obtained, similar to the 28.32% value reported by Scatolino et al. [39]. Reis et al. [40] indicated the predominance of cellulose and lignin in CP, which is important for protecting coffee seeds. Moreover, cellulose is responsible for stiffness in composites [41], and lignin provides biological and mechanical resistance along with being a hydrophobic material [39]. The available nitrogen content was found to be 2.51 ± 0.08%, similar to the value reported by Arya et al. [42] of 2.79% for coffee silver skin. The measured protein content was 2.51 ± 0.08%, and the fat content was 1.17 ± 0.12%. Murthy et al. [43] determined that CP showed the lowest values for protein and fat content compared with other coffee residues, and Iriondo-DeHond et al. [44] reported protein and fat contents of 3.1% and of 0.3%, respectively, which are different from those found in this work. The parameters evaluated in the proximal analysis were influenced by several factors such as geographical location, the age of the plant, the type of soil, and climatic conditions [41,45,46].

### 3.2. Coffee Parchment and Polymer Ribbon Characterization

#### 3.2.1. Scanning Electron Microscopy (SEM)

**Figure 1:**
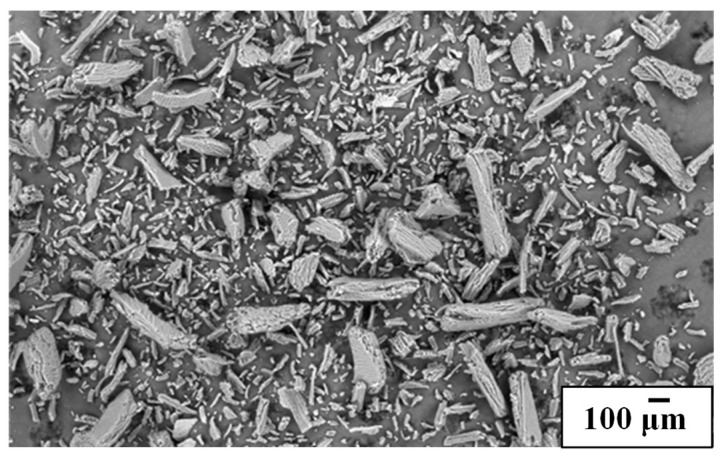
SEM micrograph of coffee parchment.

In Figure 1, the morphology of the ground CP is shown. The powder size ranged from 60 µm to 400 µm, with elongated shapes associated with the cellulose present in its structure [40].

**Figure 2:**
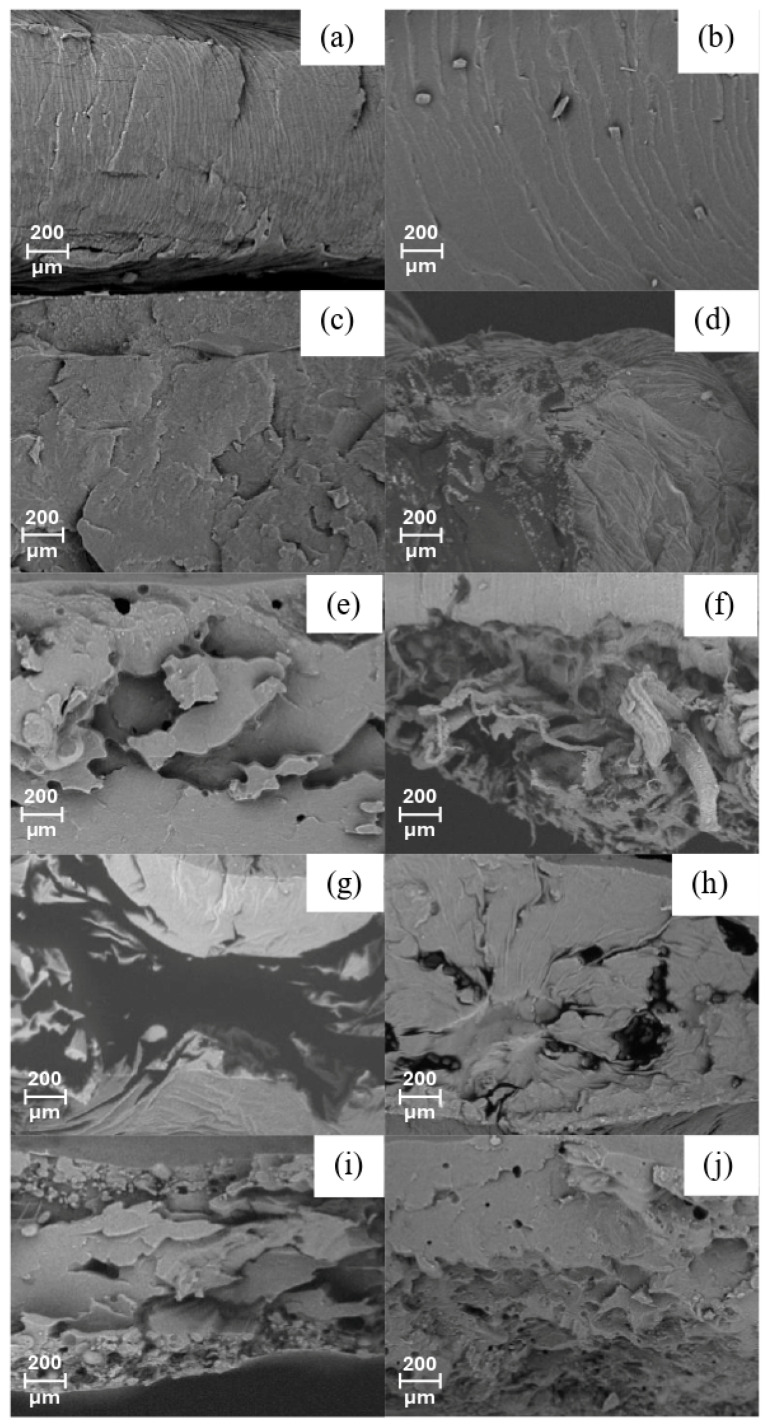
SEM micrographs of (**a**) PLA, (**b**) PBAT, (**c**) PLA PBAT, (**d**) PLA PBAT CO, (**e**) PLA PBAT CO 5% CP, (**f**) PLA PBAT CO 5% CPf, (**g**) PLA PBAT CO 7.5% CP, (**h**) PLA PBAT CO 7.5% CPf, (**i**) PLA PBAT CO 10% CP, and (**j**) PLA PBAT CO 10% CPf.

The morphology of the cross-section of the blends of neat polymers and some representative blends are shown in Figure 2, in which the influence of the CO plasticizer, CP, and functionalization can be observed. Figure 2a,b shows the neat polymers, PLA, and PBAT with a smooth surface. Figure 2c shows the micrograph for the PLA PBAT blend, with some cracks. It has been reported in the literature that PLA PBAT blends have limited miscibility [16]. After CO incorporation, the PLA PBAT CO blend showed a smooth surface morphology (Figure 2d). Plasticizers improve interfacial adhesion at the phase boundaries of the components in a blend [47]. In Figure 2e,f, the PLA PBAT CO 5% functionalized and non-functionalized blends are shown. The morphology changes from sea-island to co-continuous [48,49]. The functionalization process led to higher compatibility between the CP and the polymers [50,51]. In Figure 2f, some elongated fibrils can be seen. It has also been observed for PLA PBAT blends [27,49]. For the 7.5% CP and CPf samples, this plastic behavior was also observed (Figure 2g,h). However, for the 10% CP and CPf fibers in Figure 2i,j, small holes can be seen. These can be attributed to the pulling out of the CP particle during the cryogenic fracture [52].

#### 3.2.2. Fourier Transform Infrared Spectroscopy (FTIR)

**Figure 3:**
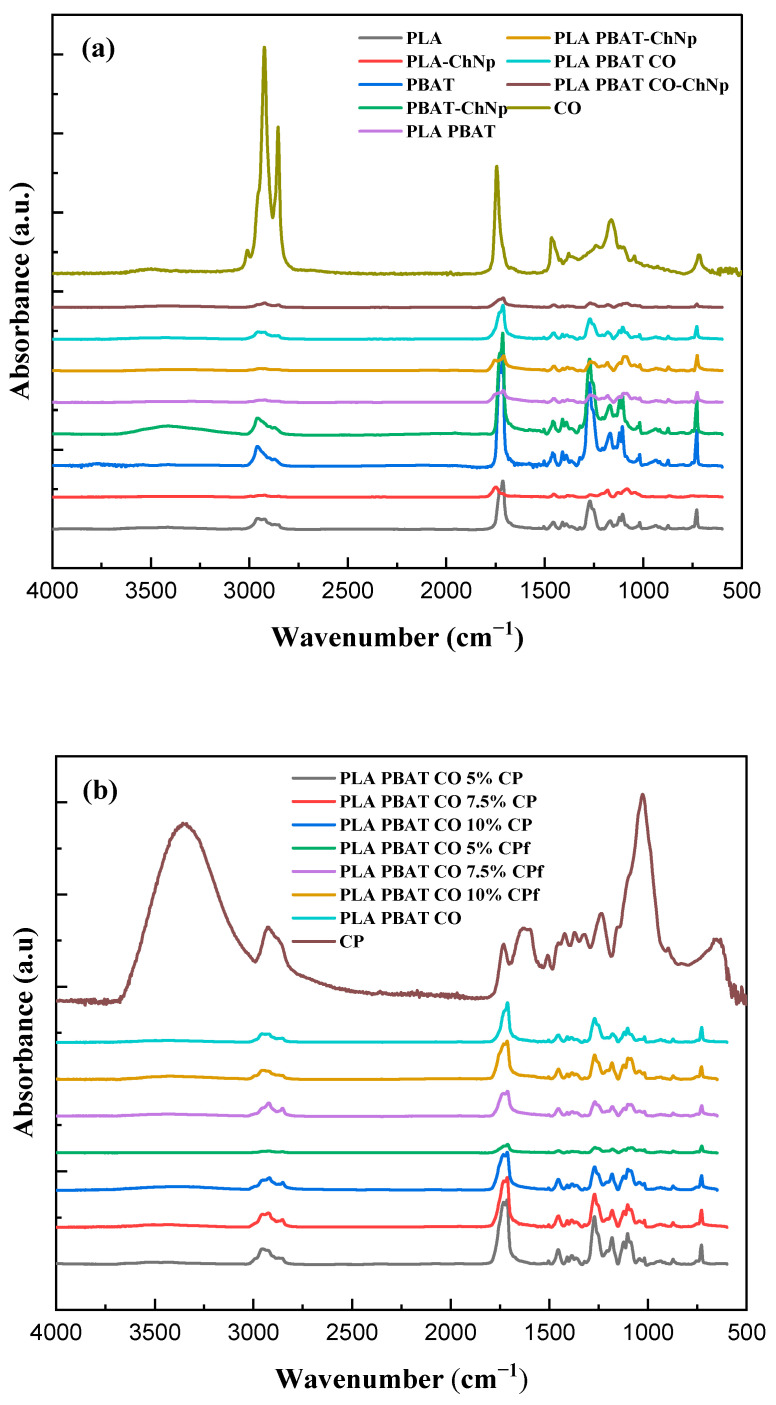
FTIR spectrum of (**a**) the neat polymers, blends and coffee oil and (**b**) coffee parchment and functionalized samples.

The FTIR spectra from Figure 3 shows the different functional groups of the CP, CO, PLA, PBAT, and blends. For the CO (Figure 3a), the ester groups together with the fatty acids were the most common functional groups reported by Raba et al. [53] and Dong et al. [54]. The main functional groups for PLA are a band between 3200 and 2700 cm^−1^ for -OH stretching and at 1726 cm^−1^ and 1286 cm^−1^ for C=O and C-O-C stretching, respectively [27,55]. In the PBAT spectrum, the -CH_2_ group from the asymmetric stretching vibration of methylene is observed between 2958 cm^−1^ and 2873 cm^−1^ [56]. Another important band present in the PBAT is the -C=O group located at 1715 cm^−1^, which is related to the asymmetric stretching vibration of ester carbonyl present on ester bonds [57]. After adding the oil to the PLA/PBAT blends, changes were seen in the PLA PBAT and PLA PBAT CO spectra, with an increase in the peaks at 3000–2800 cm^−1^ (-CH_2_ groups belonging to fatty acids and -CH stretching vibration belonging to peaks of the saturated carbon chain) and at 1800–1750 cm^−1^ (-O·C= groups belonging to triglycerides and the -C=O group belonging to the carbonyl stretching of lipid and fatty acids ester group) [54]. The coating effect of ChNp is not reflected in the FTIR spectra.

Figure 3b shows the change in the FTIR spectra due to the incorporation of different CP concentrations to the polymer blend and functionalization. The functional groups of CP were -OH between 3100 and 2800 cm^−1^, with the -CH and -CO of the acetyl group from lignin in the polymer blends located at 1024 cm^−1^ [40]. The peak between 1800 and 1700 cm^−1^ corresponded to the C=O group, which was related to the vibrations of ester carbonyl, as reported by Al-Itry et al. [58]. For the functionalization process (CPf), only a small decrease in the -OH band between 3100 and 2800 cm^−1^ was observed for the PLA PBAT CO 5% CPf compared with the non-functionalized sample [13]. For the ChNp coated samples, no changes were found (not shown).

#### 3.2.3. Thermogravimetric Analysis (TGA)

**Figure 4:**
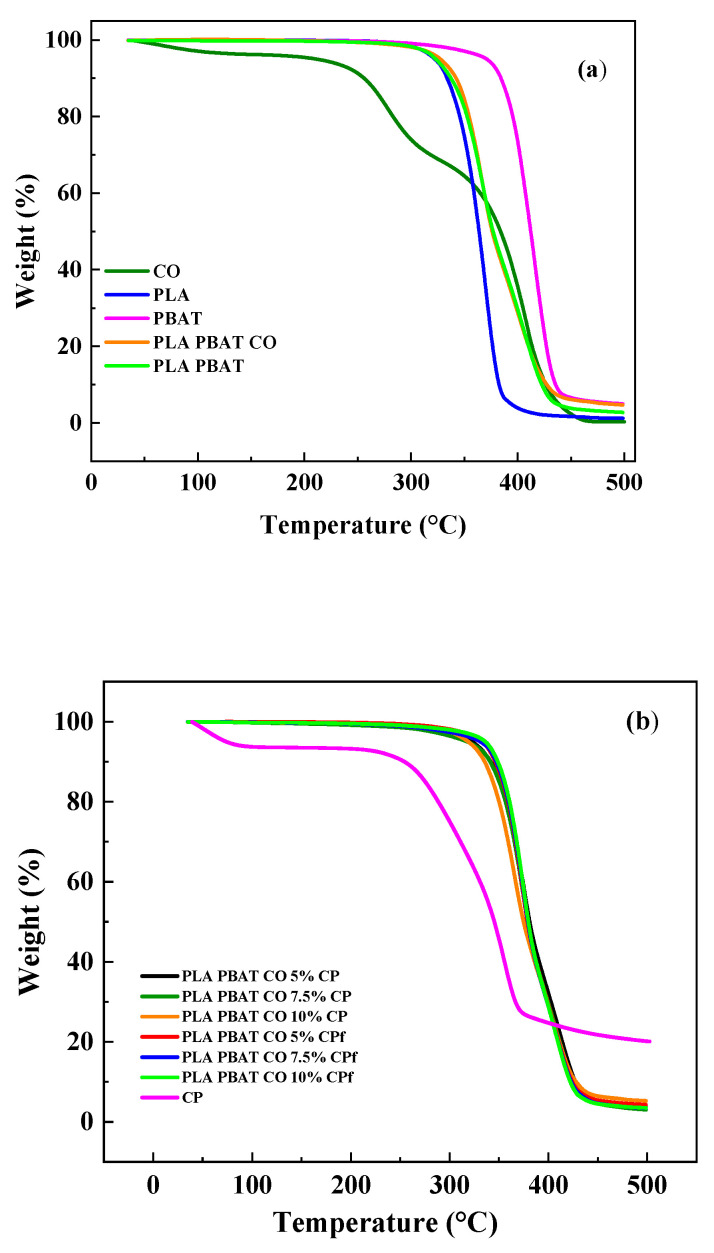

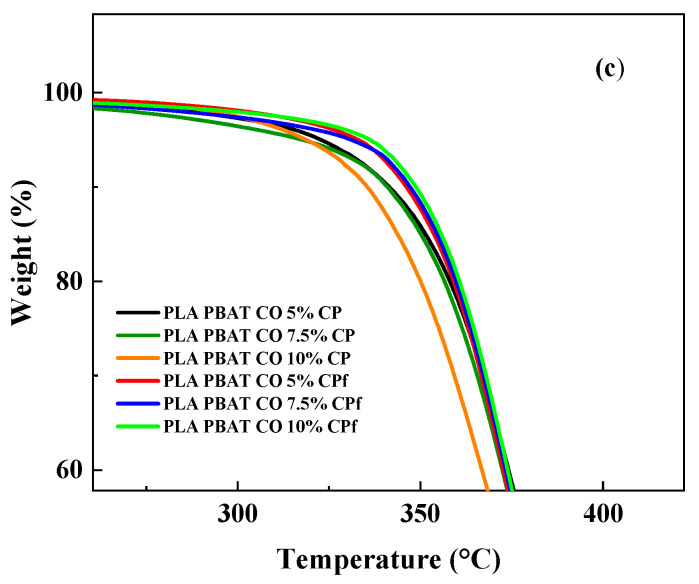
Representative TGA curves of (**a**) neat polymers, coffee oil, and polymer blends without coffee parchment and (**b**) polymer blends with different coffee parchment concentrations (5, 7.5, and 10%) with and without functionalization. (**c**) Detailed view of curve in (**b**).

Figure 4 shows the TGA analysis of the representative samples. For the CO (Figure 4a), a first decomposition stage between 220 °C and 315 °C and a second decomposition stage ranging from 315 °C to 500 °C were observed. Raba et al. [59] found similar ranges of decomposition temperatures for green coffee bean oil between 220 °C and 365 °C and between 340 °C and 525 °C. They concluded that it has thermal stability up to 216 °C and that the main weight loss is attributed to the thermal degradation of saturated and unsaturated fatty acids, the main components of the oil. The neat polymers (PLA and PBAT) showed one-step thermal decomposition. The PLA decomposition temperature started at 323 °C, and that of PBAT started at 377 °C, with their higher thermal decomposition temperatures being due to their aromatic structure compared with PLA. Wang et al. [60] reported an initial decomposition temperature of 343.8 °C for PLA and of 381 °C for PBAT, similar to the values found in this work. The PLA and PBAT blend showed intermediate behavior between PLA and PBAT. No significant effect was observed with the addition of CO.

The effect of CP incorporation and functionalization can be seen in Figure 4b. The main weight loss occurred from 250 to 372 °C. Bok et al. [61] reported a peak weight loss of 307 °C for coffee grounds. A small increase in the decomposition temperature was observed for the PLA PBAT CO 5% CPf (332 °C) compared with the PLA PBAT CO 5% CP (319 °C) sample, indicative of slightly higher thermal stability for the sample due to the functionalization process. The same behavior was observed for 7.5% and 10% CP incorporation, as can be seen in Figure 4c, with values of 325.1 °C (7.5% CP) increasing to 337.9 °C (7.5% CPf) and from 323.6 °C (10% CP) to 340 °C (10% CPf). When adding talc and diatomite to PLA PBAT blends, an increase in thermal stability was reported by Ding et al. [62]. On the other hand, an increase in the thermal decomposition temperature was found with the incorporation of different percentages of lignin (20, 40, and 50%) into poly(3-hydroxybutyrate) with microcrystalline cellulose and PLA blends by Jaffur et al. [63].

#### 3.2.4. Differential Scanning Calorimetry (DSC)

**Figure 5:**
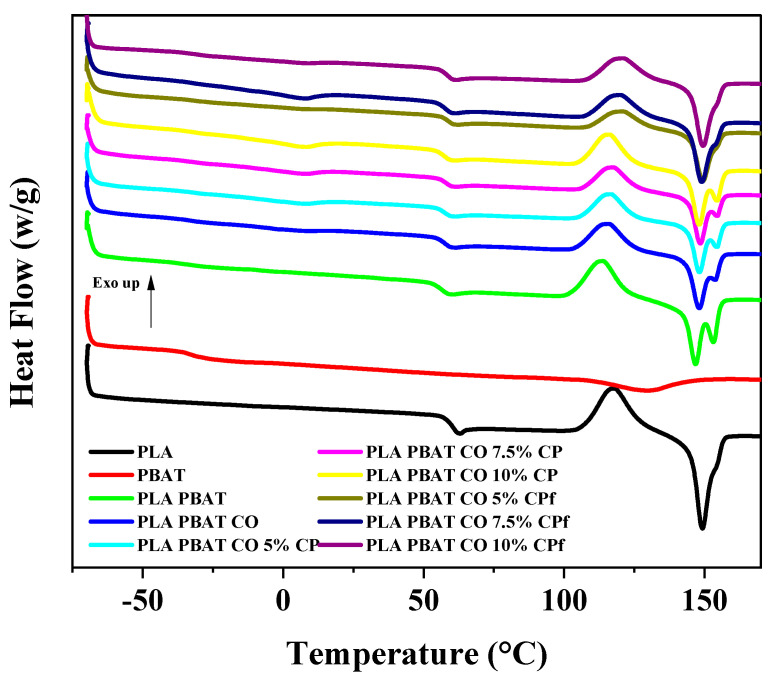
DSC thermograms of the samples.

Figure 5 shows the DSC thermograms of the representative samples. The glass transition temperature (Tg) of the PLA was about 62 °C, and for PBAT, it was about −34 °C. Values of 61 °C and −30 °C were reported by Ding et al. [62]. No significant changes in Tg were observed for the PLA PBAT, PLA PBAT CO, or blends with CP or CPf.

For PLA, the melting temperature (Tm) was about 150 °C, and for PBAT, it was about 129 °C, in agreement with those values reported by Su et al. [45] of 149.6 °C and 120.4 °C for PLA and PBAT, respectively. The PLA PBAT showed two separate Tm values corresponding to each component, as mentioned in the literature. When the components in a blend are miscible, only one endotherm is observed. On the other hand, the lack of miscibility results in the different transition temperatures corresponding to each of the components [16].

PLA showed a cold crystallization temperature (Tcc) of 117 °C located between the Tg and Tm. This is related to the crystallization of materials during heating. The same value was reported by Carbonell-Verdu et al. [64]. On the other hand, there was a decrease in Tcc for the PLA PBAT and PLA PBAT CO blends compared with PLA. For these blends, two endothermic peaks were observed because of different populations of lamellae, with the first melting peak associated with a small population and imperfect crystals and the second peak (Tm) associated with the melting of more perfect crystals [65]. Mysiukiewicz et al. [66] observed a decrease in Tcc for the PLA matrix with the addition of linseed cake with a different oil content. This was attributed to an increase in nucleation sites. Coffee oil is mainly composed of triacylglycerols similar to those found in vegetable oils [67]. Carbonell-Verdu et al. [64] found that vegetable oil derivatives act as compatibilizers and plasticizers. Coffee oil acts as a compatibilizer due to the functional groups present in its major components (mainly -OH) reacting with the carboxyl groups of PLA or PBAT, increasing miscibility. In Figure 5, a decrease in the second endothermic peak (Tm) for the PLA PBAT CO sample compared with the PLA PBAT sample was observed. Regarding plasticizing, it has been reported that their effect is not as strong as those of primary plasticizers like citrates and polyethylene glycol (PEG) [43].

On the other hand, there was an increase in the Tcc in the blends after CP incorporation. The CPf samples showed only one endothermic peak (Tm) at higher temperatures compared with PLA. This was related to the formation of more perfectly formed crystals [65]. Therefore, higher miscibility due to functionalization was achieved [16]. This was also confirmed in the SEM micrographs, in which better incorporation of the cellulose fibers into the blend was observed, especially for the PLA PBAT CO 5% CPf and PLA PBAT CO 7.5% CPf ribbons.

#### 3.2.5. Mechanical Properties

Table 3 shows the results of the mechanical properties. It is well known that PLA demonstrates brittleness, low deformability, and low impact resistance which limits its applications. To reduce brittleness and increase deformability, it is recommended to blend this with other polymers or add compatibilizers to improve the desired properties [44,68].

PLA PBAT blend was compared with the neat polymers (PLA and PBAT), finding the Young’s modulus and tensile strength between the PBAT and PLA. By adding CO, when comparing the PLA PBAT CO-ChNp and PLA PBAT CO blends, there was a decrease in Young’s modulus and the tensile strength and an increase in the elongation at break. However, the elongation was lower than that for the neat PBAT. Su et al. [45] observed a decrease of 600% for the elongation at break compared with the neat PBAT for a 50/50 PLA/PBAT ratio due to morphological changes in the blends during processing. Oils of vegetable origin in polymer blends help improve miscibility, as mentioned before [64]; they found an increase in the elongation at break by incorporating cottonseed oil into a PLA/PBAT 80/20 blend. In this work, for PLA PBAT ribbons, the elongation at break was similar to that of the PLA PBAT CO blend. However, when ChNp were incorporated as a coating, an increase in the values for the elongation at break of were obtained (from 3.89% for the PLA PBAT-ChNp ribbons compared with the PLA PBAT CO-ChNp ones (6.02%)), increasing polymer flexibility. Moreover, in these blends, there was a decrease in Young’s modulus and the tensile strength, with values from 662.96 MPa to 608.86 MPa and 18.33 MPa to 13.88 MPa, respectively. This behavior was observed in the work of Marbach and Mörbitz [51]. They evaluated the effect of epoxidized soybean oil on the mechanical properties of PLA and PBAT, resulting in a decrease in Young’s modulus and tensile strength because of the oil, which acted as a plasticizer with a homogeneous distribution between the polymers in the blend.

When CP was added, Young’s modulus decreased as the CP content as well as the tensile strength increased. Pregi et al. [16] found same behavior for lignin incorporated into PLA PBAT blends. They observed that the lignin was embedded into the PBAT without any reinforcing effect. The elongation at break was not significantly affected by the presence of ChNp or by functionalization (CPf). This can be attributed to the interfacial tension with subsequent detachment during stretching. When more CP is added, it agglomerates, affecting the mechanical properties [60]. Correa-Pacheco et al. [69] found that adding prickly pear powder to a PLA/PBAT 60/40 blend resulted in an increase in stiffness and better adhesion to the polymer. In this work, Young’s modulus and the tensile strength showed significant statistical differences with different concentrations of CP. However, for the samples with 10% CPf, there were no differences for the studied properties. Liu et al. [70] found that incorporating vinyltrimethoxysilane (VTMS)-grafted lignin into PBAT led to a decrease in the elongation at break and an increase in Young’s modulus and the tensile strength as the lignin concentration increased. On the other hand, the incorporation of PBAT into a PLA blend with thermoplastic cereal flour changed the polymer blend’s behavior from brittle to ductile. However, with the presence of thermoplastic cereal flour (33%), the elastic modulus decreased [71]. Therefore, different behavior was observed for PLA and PBAT with filler incorporation.

On the other hand, when chitosan is applied as a coating, it solidifies upon contact with a surface due to its adhesive effect, which depends on several factors, such as the concentration of chitosan, molecular weight, the type of acid in which it was solubilized, or the type of plasticizer used [72]. In this work, a ChNp coating caused a decrease in Young’s modulus and the tensile strength for the samples with CO, as mentioned before, and for the CPf-ChNp samples at 5% and 10% CP. The elongation at break did not show significant statistical differences. Some authors have reported that Young’s modulus and the tensile strength are strongly influenced by the incorporation of coffee husks and hulls compared with changes in the elongation at break [37]. Different mechanical behaviors have been found when using fillers in polymer blends. Oliaei [73] added microfibrillated lignocellulose to PCL, obtaining an improvement in tensile strength compared with the neat polymer. For neat PCL, a value of 11.5 MPa for the tensile strength was obtained. When using wood fibers at 25%, this increased to 20.6 MPa, and when using microfibrillated lignocellulose at 27%, this value was 43 MPa. This was attributed to the dispersion of wood fibers in the polymer matrix for the different particle sizes of the nanofiber, generating better interfacial adhesion between the fibers and an improvement in the mechanical properties. Another study by Sanaka and Sahu [74] reported the mechanical behavior of polyurethane with the incorporation of Ti_3_C_2_T_x_ nanofiller, finding an increase in tensile strength. However, a decrease in the elongation at break (as a percentage) with an increase in the concentration of the nanofiller was observed due to the dispersion of the nanofiller and the interfacial adhesion of the polymer matrix. In this study, with the incorporation of CP as filler, a decrease in tensile strength was found. When comparing PLA PBAT CO (15.74 ± 2.49 MPa) with PLA PBAT CO 5% CP (15.55 ± 0.57 MPa), PLA PBAT CO 7.5% CP (10.48 ± 0.73 MPa), and PLA PBAT CO 10% CP (7.37 ± 0.55 MPa), the opposite trend can be seen. This was also obtained for the functionalized samples with and without ChNp. However, for the samples with only a ChNp coating, the same behavior mentioned by Sanaka and Sahu [74] was found. Regarding the elongation at break, similar values between samples were observed, as mentioned before.

On the other hand, Sharma [75] measured the mechanical properties of polyurethane and Cloisite20A coatings, observing a partially intercalated and tactoid-like morphology with improvement in the elongation at break and toughness. However, there were decreases in the stiffness, ultimate tensile strength, and Young’s modulus, which were attributed to the agglomerates present in the composite. In this study, a general decrease in Young’s modulus was observed as the CP concentration increased, which could be associated with possible agglomerates. On the other hand, the SEM micrographs in Figure 2f,h show good miscibility of the PLA PBAT CO 5% CPf and PLA PBAT CO 7.5% CPf blends due to the functionalization process, in agreement with the mechanical property results.

**Figure 6:**
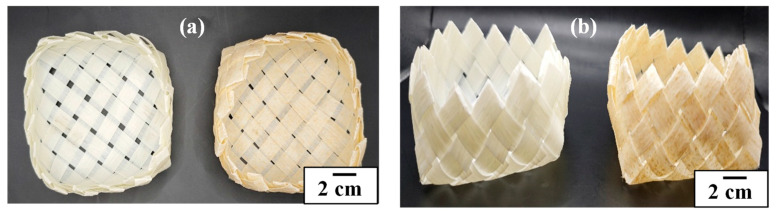
Polymer blend with coffee oil (left) and polymer blend with coffee oil and 5% coffee parchment (right) woven ribbons for biodegradable packaging: (**a**) top view and (**b**) side view.

After characterizing the ribbons, they were manually woven in a similar way to the baskets used to contain berries for use in applications such as food packaging. Figure 6 shows the biodegradable packaging woven baskets. A post-harvest study on blueberries’ shelf-life preservation is in progress.

## 4. Conclusions

In this study, extruded ribbons from PLA PBAT polymer blends incorporating CP, CO, and ChNp as a coating were characterized as an ecological alternative for environmentally friendly packaging. SEM showed the miscibility of the PLA PBAT blend with CP functionalization. FTIR analysis confirmed the presence of -OH, CO, -C-H, and -C-O functional groups in the blends. TGA showed that the samples underwent their main weight loss between 200 °C and 400 °C. DSC confirmed the miscibility, especially for CPf, for which one single peak was observed. Based on the mechanical properties, when CP was added to the blends, the elongation at break was not significantly affected by the presence of ChNp or CPf. The most affected properties were the Young’s modulus and the tensile strength. Although biodegradable packaging currently remains a challenge regarding the barrier and mechanical properties in an increasing and demanding market, this research opens a possibility for the replacement of plastics in packing and decreasing the environmental impact with the use of coffee by-products incorporated into PLA PBAT blends for the shelf-life extension of fruit and vegetables.

## Figures and Tables

**Table 1:** Ribbon’s formulations.

Nomenclature	Sample
PLA	Polylactic acid
PLA-ChNp	Polylactic acid with chitosan nanoparticle coating
PBAT	Poly (butylene adipate-co-terephthalate)
PBAT-ChNp	Poly (butylene adipate-co-terephthalate) with chitosan nanoparticle coating
PLA PBAT	Polylactic acid and poly (butylene adipate-co-terephthalate) blend
PLA PBAT-ChNp	Polylactic acid and poly (butylene adipate-co-terephthalate) blend with chitosan nanoparticle coating
PLA PBAT CO	Polylactic acid and poly (butylene adipate-co-terephthalate) blend with coffee oil
PLA PBAT CO-ChNp	Polylactic acid and poly (butylene adipate-co-terephthalate) blend with coffee oil and chitosan nanoparticle coating
PLA PBAT CO 5% CP	Polylactic acid and poly (butylene adipate-co-terephthalate) blend with coffee oil and 5% coffee parchment
PLA PBAT CO 5% CP-ChNp	Polylactic acid and poly (butylene adipate-co-terephthalate) blend with coffee oil, 5% coffee parchment, and chitosan nanoparticle coating
PLA PBAT CO 7.5% CP	Polylactic acid and poly (butylene adipate-co-terephthalate) blend with coffee oil and 7.5% coffee parchment
PLA PBAT CO 7.5% CP-ChNp	Polylactic acid and poly (butylene adipate-co-terephthalate) blend with coffee oil, 7.5% coffee parchment, and chitosan nanoparticle coating
PLA PBAT CO 10% CP	Polylactic acid and poly (butylene adipate-co-terephthalate) blend with coffee oil and 10% coffee parchment
PLA PBAT CO 10% CP-ChNp	Polylactic acid and poly (butylene adipate-co-terephthalate) blend with coffee oil, 10% coffee parchment, and chitosan nanoparticle coating
PLA PBAT CO 5% CPf	Polylactic acid and poly (butylene adipate-co-terephthalate) blend with coffee oil and 5% functionalized coffee parchment
PLA PBAT CO 5% CPf-ChNp	Polylactic acid and poly (butylene adipate-co-terephthalate) blend with coffee oil, 5% functionalized coffee parchment, and chitosan nanoparticle coating
PLA PBAT CO 7.5% CPf	Polylactic acid and poly (butylene adipate-co-terephthalate) blend with coffee oil and 7.5% functionalized coffee parchment
PLA PBAT CO 7.5% CPf-ChNp	Polylactic acid and poly (butylene adipate-co-terephthalate) blend with coffee oil, 7.5% functionalized coffee parchment, and chitosan nanoparticle coating
PLA PBAT CO 10% CPf	Polylactic acid and poly (butylene adipate-co-terephthalate) blend with coffee oil and 10% functionalized coffee parchment
PLA PBAT CO 10% CPf-ChNp	Polylactic acid and poly (butylene adipate-co-terephthalate) blend with coffee oil, 10% functionalized coffee parchment, and chitosan nanoparticle coating

**Table 2:** Proximal analysis of coffee parchment.

Type of Analysis	Value (%)
Moisture	3.25 ± 0.17
Ash	0.71 ± 0.03
Cellulose	53.09 ± 3.42
Lignin	23.60 ± 1.74
Available nitrogen	2.51 ± 0.08
Protein	2.51 ± 0.08
Fat	1.17 ± 0.12

**Table 3:** Mechanical properties of the different blends: Young’s modulus, tensile strength, and elongation at break.

Sample	Young’s Modulus (MPa)	Tensile Strength (Mpa)	Elongation at Break (%)
PLA	1446.96 ± 93.78 ^h^	43.66 ± 3.49 ^h^	5.05 ± 0.54 ^a^
PLA-ChNp	1388.11 ± 116.53 ^h^	50.71 ± 1.51 ^i^	17.41 ± 7.48 ^b^
PBAT	79.46 ± 2.69 ^a^	7.59 ± 0.40 ^b^	50.84 ± 9.59 ^c^
PBAT-ChNp	77.02 ± 0.46 ^a^	7.62 ± 0.33 ^b^	62.78 ± 16.39 ^d^
PLA PBAT	636.22 ± 74.31 ^ef^	15.78 ± 1.79 ^f^	4.08 ± 0.63 ^a^
PLA PBAT-ChNp	662.96 ± 44.75 ^f^	18.33 ± 3.58 ^g^	3.89 ± 0.56 ^a^
PLA PBAT CO	766.40 ± 161.49 ^g^	15.74 ± 2.49 ^f^	3.01 ± 0.41 ^a^
PLA PBAT CO-ChNp	608.86 ± 89.89 ^def^	13.88 ± 0.85 ^de^	6.02 ± 3.94 ^a^
PLA PBAT CO 5% CP	670.74 ± 47.72 ^f^	15.55 ± 0.57 ^f^	3.53 ± 0.26 ^a^
PLA PBAT CO 5% CP-ChNp	678.97 ± 23.57 ^f^	19.11 ± 1.21 ^g^	4.38 ± 0.23 ^a^
PLA PBAT CO 7.5% CP	590.72 ± 37.04 ^de^	10.48 ± 0.73 ^c^	2.69 ± 0.12 ^a^
PLA PBAT CO 7.5% CP-ChNp	794.49 ± 40.03 ^g^	18.45 ± 0.93 ^g^	3.71 ± 0.41 ^a^
PLA PBAT CO 10% CP	477.28 ± 43.61 ^c^	7.37 ± 0.55 ^b^	2.19 ± 0.15 ^a^
PLA PBAT CO 10% CP-ChNp	681.15 ± 27.48 ^f^	19.63 ± 0.94 ^g^	4.16 ± 0.28 ^a^
PLA PBAT CO 5% CPf	775.98 ± 34.07 ^g^	15.17 ± 0.74 ^ef^	3.19 ± 0.18 ^a^
PLA PBAT CO 5% CPf-ChNp	615.43 ± 49.79 ^def^	12.99 ± 1.26 ^d^	3.53 ± 0.20 ^a^
PLA PBAT CO 7.5% CPf	397.48 ± 66.09 ^b^	4.80 ± 0.75 ^a^	2.06 ± 0.31 ^a^
PLA PBAT CO 7.5% CPf-ChNp	563.97 ± 26.04 ^d^	12.88 ± 0.54 ^d^	3.42 ± 0.18 ^a^
PLA PBAT CO 10% CPf	497.83 ± 31.67 ^c^	7.53 ± 0.28 ^b^	2.44 ± 0.17 ^a^
PLA PBAT CO 10% CPf-ChNp	464.41 ± 66.11 ^c^	6.20 ± 1.22 ^ab^	2.06 ± 0.14 ^a^

Mechanical properties of different ribbons compositions. Young’s modulus ANOVA with Duncan’s multiple range test (means ± SD): F = 199.43, gl = 140, and *p* < 0.0001. Tensile strength ANOVA with Duncan’s multiple range test (means ± SD): F = 461.87, gl = 140, and *p* < 0.0001. Elongation at break ANOVA with Duncan’s multiple range test (means ± SD): F = 101.60, gl = 140, and *p* < 0.001. Different letters mean there were significant differences. PLA = polylactic acid, PBAT = poly (butylene adipate-co-terephthalate), CO = coffee oil, ChNp = nanostructured chitosan coating, CP = coffee parchment, and CPf = coffee parchment with functionalization process.

## Data Availability

The original contributions presented in the study are included in the article, further inquiries can be directed to the corresponding authors.

## Abbreviations

The following abbreviations are used in this manuscript:

CP	Coffee parchment
CO	Coffee oil
ChNp	Chitosan nanoparticles
PLA	Polylactic acid
PBAT	Poly(butylene adipate-co-terephthalate)
CPf	Coffee parchment with the functionalization process
SEM	Scanning electron microscopy
TGA	Thermogravimetric analysis
DSC	Differential scanning calorimetry
FTIR-ATR	Confocal micro-Raman spectroscopy equipment coupled with Fourier transform infrared-attenuated total reflection
Iw	Initial sample weight
Fw	Final sample weight
AOAC	Asociation of Analytical Communities
AACC	American Association of Cereal Chemists
SW	Sample weight
CWS	Crucible weight with sample
ECW	Empty crucible weight
AW	Ash weight
CWS	Crucible weight with sample
DWR	Dry weight of the residue
WCM	Weight of cellulosic material
LW	Lignin weight
DSW	Dry sample weight
F	Protein factor
N	Titration normality
mN	Milliequivalents of nitrogen
DMTA	Dynamic mechanical thermal analysis
FW	Fat weight
WCF	Weight of container with fat
ECW	Empty container weight
